# Experimental inheritance of antibiotic acquired dysbiosis affects host phenotypes across generations

**DOI:** 10.3389/fmicb.2022.1030771

**Published:** 2022-12-01

**Authors:** Vienna Kowallik, Ashutosh Das, Alexander S. Mikheyev

**Affiliations:** ^1^Okinawa Institute of Science and Technology, Tancha Onna-son, Okinawa, Japan; ^2^Australian National University, Canberra, ACT, Australia; ^3^Chattogram Veterinary and Animal Sciences University, Khulshi, Chattogram, Bangladesh

**Keywords:** microbiome, antibiotics, honey bees, experiments, dysbiosis, transgenerational effects

## Abstract

Microbiomes can enhance the health, fitness and even evolutionary potential of their hosts. Many organisms propagate favorable microbiomes fully or partially *via* vertical transmission. In the long term, such co-propagation can lead to the evolution of specialized microbiomes and functional interdependencies with the host. However, microbiomes are vulnerable to environmental stressors, particularly anthropogenic disturbance such as antibiotics, resulting in dysbiosis. In cases where microbiome transmission occurs, a disrupted microbiome may then become a contagious pathology causing harm to the host across generations. We tested this hypothesis using the specialized socially transmitted gut microbiome of honey bees as a model system. By experimentally passaging tetracycline-treated microbiomes across worker ‘generations’ we found that an environmentally acquired dysbiotic phenotype is heritable. As expected, the antibiotic treatment disrupted the microbiome, eliminating several common and functionally important taxa and strains. When transmitted, the dysbiotic microbiome harmed the host in subsequent generations. Particularly, naïve bees receiving antibiotic-altered microbiomes died at higher rates when challenged with further antibiotic stress. Bees with inherited dysbiotic microbiomes showed alterations in gene expression linked to metabolism and immunity, among other pathways, suggesting effects on host physiology. These results indicate that there is a possibility that sublethal exposure to chemical stressors, such as antibiotics, may cause long-lasting changes to functional host-microbiome relationships, possibly weakening the host’s progeny in the face of future ecological challenges. Future studies under natural conditions would be important to examine the extent to which negative microbiome-mediated phenotypes could indeed be heritable and what role this may play in the ongoing loss of biodiversity.

## Introduction

The Anthropocene provides many novel selection pressures on organisms, such as climate change and the application of agrochemicals and antibiotics (Sánchez-Bayo and Tennekes, 2017; Cavicchioli et al., 2019). Organisms respond in various ways to these pressures, ranging from the evolution of resistance to extinction. When animals are exposed to nutritional disturbance (e.g., by chemicals), in addition to potential direct effects on the organism itself, their gut microbiome may be affected. Dwelling at the interface between host epithelia and the external environment, microbial symbionts (microbiomes) can affect host health by influencing traits such as nutrition, immunity and behavior (Round and Mazmanian, 2009; Flint et al., 2012; Tremaroli and Bäckhed, 2012). Microbial communities can change rapidly in composition or in gene-expression patterns when responding to ecological forces. Therefore, a microbiome can extend host evolutionary potential and may facilitate rapid host acclimation to environmental change (Alberdi et al., 2016; Henry et al., 2021). Specific gut microbial communities can provide hosts with novel functions, such as mediating insecticide resistance (Kikuchi et al., 2012; Wang et al., 2020) or promoting tolerance to thermal stress (Zare et al., 2018; Zhang et al., 2019; Raza et al., 2020). Such microbial rescue effects have the potential to stabilize host dynamics and may explain population persistence in changing environments (Mueller et al., 2020). Due to the wide range of functional benefits they provide, microbiomes are often tightly curated by the host, for example by management and vertical transmission between generations (Foster et al., 2017; Rosenberg and Zilber-Rosenberg, 2021). In general, transmission of microbiomes across generations will transmit the community and its associated functions – which may be positive or negative for the host depending on the conditions.

Indeed, a microbiome is not always beneficial for the host. Some organisms even completely lack it (Hammer et al., 2019) and the functional benefit provided by a microbiome may also be dependent on environmental conditions. For example experiments in mice show that adapted microbiomes efficiently harvest energy from food but causing obesity in recipient individuals when being transferred (Turnbaugh et al., 2006). While such efficiency may be beneficial under food restriction, it could lead to health problems in times of plenty. Importantly, evolved cooperation between hosts and symbionts can result in wide reciprocal functional inter-dependencies. In such cases, disturbances to the microbiome can compromise host health and development by, e.g., loss of important microbiome-mediated functions, or microbial production of harmful substances as a response to environmental change (Littman and Pamer, 2011; Soen, 2014). As a result, vertical transfer of such sub-optimal microbiomes could compromise host health transgenerationally. Hypothetically, in extreme cases, a host population that is unable to escape a mal-adapted microbiome may face extinction.

Dysbiotic (defined by a loss of beneficial microbes, expansion of pathobionts or loss of diversity of the healthy, homeostatic gut condition (Petersen and Round, 2014)) parental microbiomes can affect the microbiome composition and phenotypes of offspring across systems. For example, female mice inoculated with antibiotic-disturbed microbiomes will transfer this dysbiosis to the offspring causing enhanced colitis (Schulfer et al., 2018). In fish, chemical exposure causes dysbiosis which persists in F1 offspring with correlating intestinal problems (Chen et al., 2018) and even result in alterations in the F2 intestinal epigenome, transcriptome and morphology (Guzman, 2021). Diet induced microbiome changes modulate transgenerational cancer risk in mice (Poutahidis et al., 2015). In addition, another interesting study in flies showed antibiotic-mediated depletion of a commensal bacterial genus can cause non-Mendelian, transgenerational inheritance of a stress-induced phenotype (Fridmann-Sirkis et al., 2014).

By their design, antibiotics pose particular threats to microbiomes. Antibiotic pollution is omnipresent in ecosystems due to heavy usage in medicine and agriculture (Kraemer et al., 2019) and they are known to decrease microbial diversity, to compromise host-microbiome interactions, to weaken immune system homeostasis (Modi et al., 2014) and impair colonization resistance (Bäumler and Sperandio, 2016). Still so far, the focus in most studies on stress factor effects on microbiomes usually lays on immediate effects during an individual’s life (Francino, 2016), and in such cases direct effects of stressors on the host cannot clearly be disentangled from indirect effects *via* a disturbed gut microbiome.

Here, we set out to examine whether the deleterious effects of a disrupted microbiome can persist transgenerationally, using honey bees as a tractable model system. Honey bee microbiomes are socially transmitted between worker ‘generations’, whereby newly eclosed workers acquire microbiomes from their colony-mates and the direct hive environment. While this is a different vertical transmission approach from the classical parent-to-offspring one, it was successfully leading to strong co-evolution between corbiculate bees and their microbiomes (Koch et al., 2013; Kwong et al., 2017). The adult honey bee microbiome consists of ~8 bacterial phylotypes that are involved in key biological functions such as nutrition, digestion, and immunity (Engel et al., 2016; Emery et al., 2017; Kešnerová et al., 2017; Raymann and Moran, 2018). Because young adults emerge from pupation without a microbiome, they can reliably be inoculated with a microbiome of choice in the lab (Powell et al., 2014; Zheng et al., 2018; Kowallik and Mikheyev, 2021). Thus, it is possible to serially transfer microbiomes across worker ‘generations’ to study how microbial changes in response to environmental stressors affect host phenotypes and health. In addition, honey bees are important pollinators and are exposed to diverse chemicals in the agricultural landscape as well as by beekeepers. It could be shown that antibiotics have strong effects on the honey bee microbiome (Powell et al., 2021; Tian et al., 2012; Moullan et al., 2015; Li et al., 2017; Raymann et al., 2017; Baffoni et al., 2021; Jia et al., 2022) and that such dysbiosis can even be experimentally transferred between workers (Jia et al., 2022).

In our study we used controlled lab experiments passaging microbiomes affected by antibiotics from one worker cohort to the next and examined mediated effects on host physiology by exposing naïve bees receiving these microbiomes to high levels of antibiotic stress. This design allowed us to isolate changes in the microbiome from host responses and from environmental changes. We found that the microbiome was disturbed after antibiotic exposure leading to compositional and functional changes. These were both transmitted to subsequent host generations, leading to some changes in host gene expression and to high mortality under stress.

## Materials and methods

To test how honey bee microbiomes respond under antibiotic pressure and how this affects host phenotypes across generations, we conducted experiments in which microbiomes were transferred over two host cycles (worker “generations”) under sub-lethal chemical administration. In the third cycle, to examine whether past chemical exposure affects host survival, we applied lethal levels of the chemicals to which prior “generations” had been exposed. We quantified changes in both host gene expression and microbial composition using RNA-seq and 16S amplicon sequencing, respectively.

### Experimental setup

**Figure 1:**
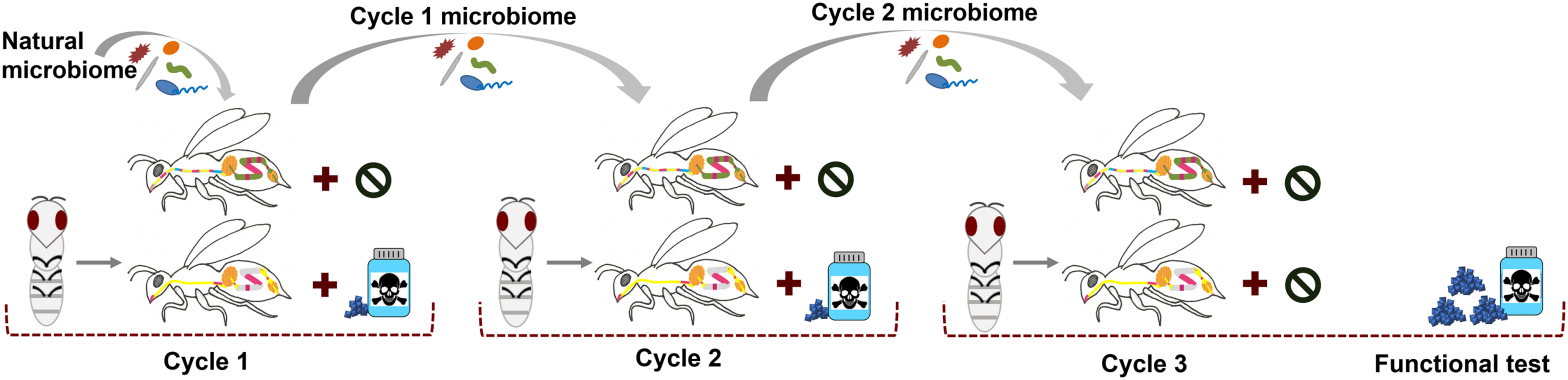
Design of the main experiment. Pupae emerge in the lab and are first inoculated for 48 h with a natural microbiome from hive siblings. Three cages per treatment were used. Throughout cycle 1 and 2, bees are continuously fed with sterile pollen and sucrose containing tetracycline or not (control). These exposed and control microbiome communities get passed to the following cycle of lab-emerged bees (cage to cage transfer). In cycle 3, bees that received control or pre-exposed microbiomes are kept naïve toward the chemical until they are administered high doses of tetracycline at the end.

The first experiment (Figure 1) was conducted in February/March 2019 at Australian National University in Canberra, Australia. See also the Supplementary information for more methodological details. The same, chemically untreated *Apis mellifera ligustica* colony was used throughout the whole experiment to avoid host genetic background changes. We started with a cohort of microbiome depleted individuals of the same age in each cycle. Late-stage pupae (dark eyes but lacking movement) were carefully removed from brood frames and allowed to develop under sterile conditions in the lab. Workers eclosing within 24 h were randomly distributed into six cages (three independent cages per treatment with ~25 bees/cage) and provided with filter-sterilized 0.5 M sucrose solution (Supplementary Figure S1). When all bees were distributed, the sucrose feeders were replaced with sterile sucrose or antibiotic-infused sucrose. We used a tetracycline hydrochloride concentration previously published in a honey bee microbiome study (450 μg tetracycline / mL sucrose (Raymann et al., 2017)). Concurrently, 10 nurse bees from the same hive were surface sterilized, and their dissected hindguts were macerated in 1:1 PBS/sucrose solution, mixed with gamma-irradiated bee bread (previously collected from colonies from the same apiary and then sterilized with 35kGY) and equally distributed across all six cages. On the following day, the remaining food was discarded and the microbiome feeding method was repeated for a second time for 24 h using again 10 nurse bee guts. On both days, small amounts of the microbiome pools were kept for later determination of the start microbiome. After the inoculation period the bees received sterile pollen and sucrose with or without antibiotics. Daily, the tetracycline solution was freshly prepared, dead bees were removed and fresh sucrose and sterile bee bread were offered *ad libitum*. Bees were maintained under these conditions for 6 days in cycle one and 10 days in cycle two, differences due to the need to have enough pupae of the same age and hive background ready for the next cycle. However, the aim was to provide enough time that the microbiome can be fully established. We previously experienced that when newly emerged bees receive a microbiome pool for 48 h, they show the full adult bee microbiome in composition and abundance after 7 days (Kowallik and Mikheyev, 2021). It is also known that under natural conditions, adult bees get colonized within the first 2 days after emergence which is followed by rapid establishment within 4 to 6 days post-eclosion (Powell et al., 2014). We therefore gave a minimum of 6 days to allow inoculation, internal growth and establishment of the microbiome. For microbiome transfer in cycle two the newly emerged bees received the microbiome from the previous cycle to mimic generation-spanning microbiome transmission. For this, three bees in each cage were sacrificed, surface sterilized, and their dissected hindguts were mixed with sterile pollen and administered to one bee cage of the next cycle for 48 h (cage to cage transfer provided three independent cage replicates per treatment). We always kept small amounts of these transfer pools for later sequencing. All other surviving bees in each cage at the end of cycle 1 and 2, as well as small amounts of the gut transfer pools were snap-frozen in liquid nitrogen and stored in a − 80° C freezer until further processing. In the beginning of the third cycle, control and exposed microbiomes from the previous cycle were transferred again to newly emerged bees as stated above. However, in cycle three, all cages received sterile food without toxins for 6 days. On day six, three individuals per cage were collected and snap frozen to examine the established microbiome community (“cycle 3 before stress”) at this time point. Subsequently, all cages were then challenged with a high dose of the stressor (20 mg tetracycline per mL sucrose), a concentration identified to cause 50% mortality in 24 h (LD50) during a pilot study (see Supplementary Methods). Due to the high mortality in the “exposed microbiome” cages, we counted survival after 20 h, with the surviving bees (“cycle 3 after stress”) being snap-frozen and stored at −80° C until further extractions.

We calculated the survival proportion for each day of the experiment before high stress application and plotted the mean of the three cages for both treatments for each cycle with standard deviations. To compare the control and tetracycline treatment we performed two-sided Fisher’s exact tests on alive/dead count data of the three cages for each day. For statistical analysis of the final survival data after high stress application, we used a Bayesian logistic regression approach to examine effects of past chemical exposure on survival in the face of lethal stress levels. To account for between-cage heterogeneity within treatments, we first estimated mortality levels for each cage regardless of treatment (survival ~ cage) using the *brms* package (Bürkner, 2017). We chose standard minimally informative priors and verified adequate model performance using diagnostic plots and statistics provided by the package. We then tested the hypothesis that cage mortality coefficients were the same in control vs. experimental treatment, using the *brms* hypothesis function, which computes the posterior distribution of the difference between Bayes factor levels in the contrast. This approach parallels planned linear contrasts in regression analysis. In addition, we conducted a non-parametric analysis using two-sided Fisher’s exact tests on alive/dead count data (altogether 53 control-gut and 47 tetracycline-gut individuals).

### Mechanisms underlying phenotypic effects of tetracycline-exposed microbiome transfer

**Figure 2:**
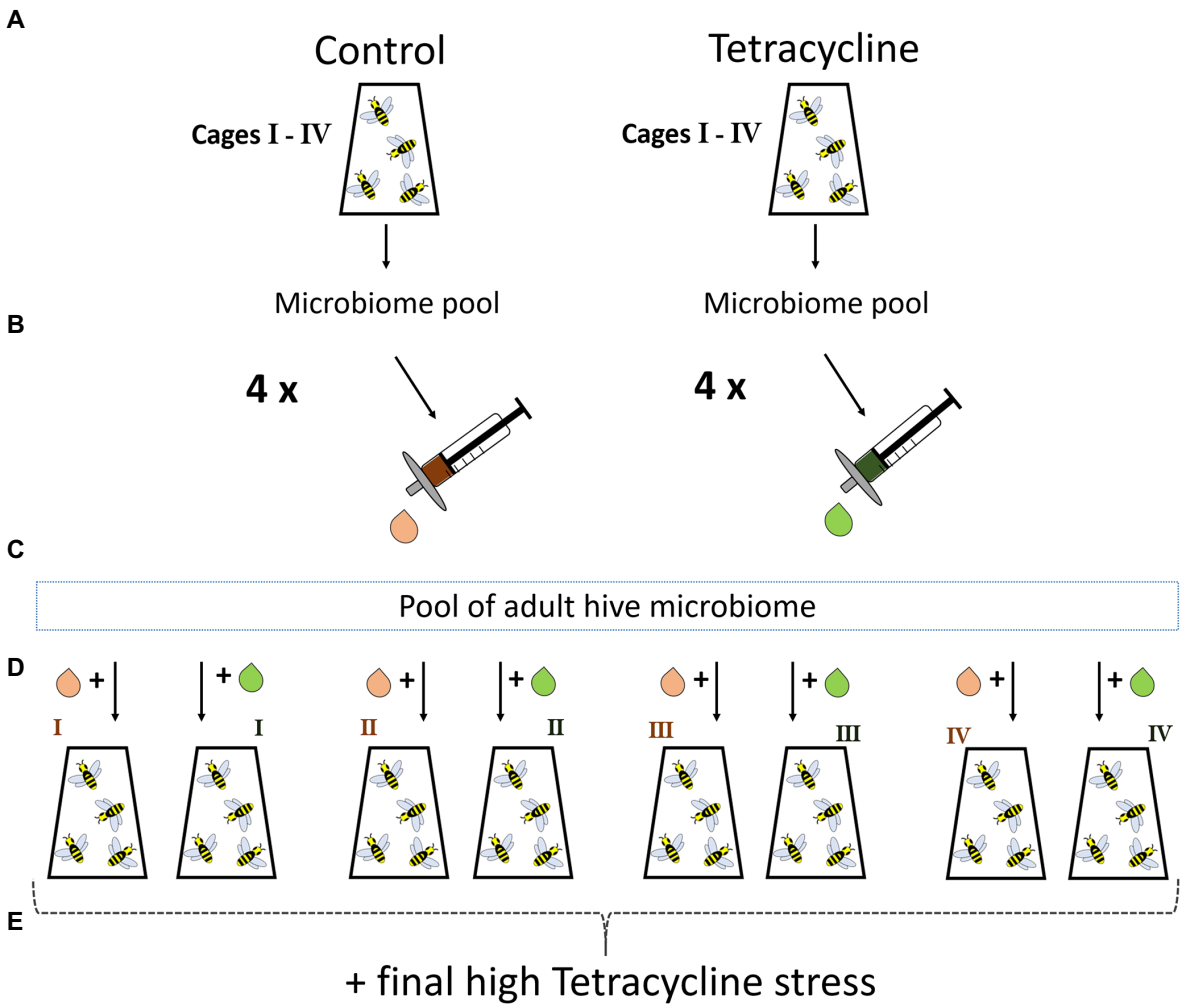
Control experiment with filtered gut solution. Emerged bees with transferred natural nurse microbiome are raised in four cages with control or tetracycline diet for 6 days **(A)**. On day six a bee gut pool for each cage is prepared as done in the first experiment and filtered to exclude microbes but to keep all potential tetracycline and derivates potentially present in the guts **(B)**. A microbiome pool of hive sibling guts is generated to allow a healthy background microbiome for newly emerged bees for the next cycle **(C)**. This microbiome pool is equally split and each part gets mixed with the filtrate of one control or tetracycline cage **(D)**. The bees receive sterile food for 6 days and are exposed to high tetracycline stress in the end and mortality is recorded **(E)**.

To exclude leftover tetracycline or derived by-products inside the transferred guts as proximal drivers of stress-induced mortality we ran an additional control experiment. In March 2021 in Okinawa Japan, we started the experiment as described before by grafting pupae. Experimental procedures were generally identical to the previous experiment. After sterile emergence, bees were distributed equally to eight cages with ~28 bees each. Microbiome transfer from nurse bees of the same hive was done as before. Four cages received tetracycline and the other sterile food only. After 6 days, the volume of four macerated guts (one more to account for any loss in the filter) per cage was filtered using a 0.2 um syringe filter to remove microbial cells. After surface-sterilizing and dissecting 20 nurse bees from the same colony, we pooled the hindguts to receive a healthy microbiome pool as base for the next cycle’s bees. This pool was equally split into eight parts, and each got mixed with the filtered gut solution of one cage from cycle 1 (Figure 2). For the next cycle, this resulted in four cages of microbiome + filtered control (supernatant of cycle 1 bee guts receiving sterile food) and four cages of microbiome + filtered tetracycline-exposed (supernatant of cycle 1 tetracycline-exposed guts) solution. All bees received sterile food for 6 days and high tetracycline dose on day six. After 15 h, mortality was recorded. The same statistical approach as described above was used by applying Bayesian logistic regression and Fisher’s exact tests (N = 4 cages; altogether 60 control-filter-gut and 50 tetracycline-filter-gut individuals).

### Extractions and sequencing

For extractions of bees from the first experiment we used the Qiagen AllPrep PowerFecal DNA/RNA Kit on abdomens of frozen bees. Every bee was first rinsed with ethanol and three subsequent rinsing steps in sterile water to clean the surfaces and then the whole abdomen or the microbiome transfers were processed following the recommended settings of the protocols, including bead beating using the Geno/Grinder®. DNA was eluted in 30 μl TE buffer. For 16S sequencing we examined the microbial community composition of 75 samples. These were one sample of start microbiome composed of the nurse microbiome pool (day 1 and day 2 pooled together), six nurse bees from the same hive as natural controls, two ZymoResearch Mock DNA controls, 12 microbiome transfer pools (one for each cage being composed of three pooled guts) for the cycle to cycle microbiome transfers in the beginning of cycle 2 and 3 (=24 pools together). In addition, we sequenced 54 individual bee abdomen from four different time points during the experiment (end of cycle 1 (9 control, 3 tetracycline), end of cycle 2 (9 control, 9 tetracycline), cycle 3 before high tetracycline application (9 control, 9 tetracycline) and after (8 control, 1 tetracycline)). We aimed to sequence three individuals per cage and time point, however, as the number of sampled individuals relied on the numbers of bees surviving, minus the ones used for gut transfer and sometimes a dissection may have gone wrong or a bee escaped, we ended up with fewer numbers of sequenced samples in some cases.

DNA of samples was submitted to DNA Sequencing Section at the Ramaciotti Centre for Genomics in Sydney Australia. Library preparation was performed based on Illumina protocol with 25-μl reactions. Illumina barcoded primers (Klindworth et al., 2013) were used to create a single amplicon of approximately 460 bp encompassing the V3-V4 region of bacterial 16S rRNA. Samples were pooled to equimolar concentration and sequenced on Illumina MiSeq v3 2 × 300 bp platform. Reads were demultiplexed on the basis of barcode sequences, allowing for one mismatch.

### 16S amplicon sequence analysis

Demultiplexed reads were processed using QIIME2 version 2019.1 (Bolyen et al., 2019), denoising of the fastq files was performed using the denoise-paired command from the DADA2 software package (Callahan et al., 2016), wrapped in QIIME2, including removal of chimeras using the “consensus” method. Decreased quality scores (below 20) of the sequences at the beginning to remove primers and end were truncated (trim-left-*f* = 17, trim-left-r = 21, trunc-len-*f* = 275, trunc-len-r 225). This resulted in a remaining overlap of ~40 bases in merged sequences. The result is an amplicon sequence variant (ASV) table, a higher-resolution analog of the traditional OTU table. For taxonomic assignment, the QIIME2 q2-feature-classifier plugin (Bokulich et al., 2018) and the Naïve Bayes classifier (Wang et al., 2007), which we trained with our primers previously, were used on the SILVA release 132 (Quast et al., 2013; Yilmaz et al., 2014).

All following graphical and statistical comparisons were performed in R using the phyloseq package (McMurdie and Holmes, 2013). In short, we first removed all non-bacterial sequences, mitochondrial and chloroplast sequences, and ASVs not present in any sample (likely artifacts) from the datasets using the *subset_taxa* and *prune_taxa* functions. We plotted rarefaction curves of all samples using the ranacapa function *ggrare* (Kandlikar et al., 2018) on the minimum sample depths (12,351 reads). Alpha diversity of the rarefied samples was explored by plotting Observed species numbers and Shannon’s diversity index. Pairwise, two-sided Wilcoxon rank sum tests were used to test for significant alpha diversity differences between treatments in each cycle. As rarefying sample counts is not recommended, unless necessary, (McMurdie and Holmes, 2014) we converted data to proportions for normalization purposes. On these proportions, non-metric Multidimensional Scaling (NMDS) was performed on Bray-Curtis distances for ordination plots.

To test for variation within groups, we used the *betadisper* function in the Vegan package, version 2.5–5 in R on the Bray-Curtis distance matrix on proportion data to calculate distances to group centroids per treatment for each cycle. Subsequently, output was plotted as ordination for visualization and *permutest* was run for each cycle to check for homogeneous distribution of samples across the two treatments. Multifactor permutational multivariate analysis of variance (PERMANOVA) on Bray-Curtis distances with 999 permutations using the ADONIS function were performed to test for effects of experimental factors on the gut community. As we sequenced single bees as well as microbiome transfers after each cycle, we first tested whether there is a difference according to method for each treatment. We also tested for cage effects in the data set in each cycle and treatment. In addition, we compared each treatment against the respective controls for all 3 cycles. Finally, we tested whether the microbiomes of each treatment changed across cycles. For taxonomic visualization we plotted the relative abundances of all genera accounting for at least 1% of the abundance across treatments and cycles. We then extracted the seven dominant taxa from the rarefied sample set and plotted their individual, total abundances across cycles with subsequent two-sided Wilcoxon rank sum tests between treatments and the respective controls. To further investigate response variation in species as well as ASV level (also see Supplemental results for more details), we pooled all cycles after checking that no cycle-specific differences could be observed and extracted the abundant species for each core genus (>1,000 reads) and plotted their abundances across the two treatments. We used online megablast against the full NCBI Nucleotide collection database on abundant ASVs (>1,000 reads) for each genus for better taxonomic resolution (sequences and alignment output in supplements). Similarly, we also plotted the total abundances of ASVs across the two treatments.

### RNA-sequencing and analysis

To understand the molecular basis of physiological effects that the microbiome’s antibiotic treatment history has on hosts, we conducted RNA-sequencing of six honey bees in cycle 3 before high stress application. We sequenced one individual per cage (three per treatment), comparing bees with tetracycline-stressed and control microbiomes.

For RNA library preparation, the QIAseq® Stranded mRNA Select Kit was used following the standard protocol. Sequencing was done on a Nextseq 2000 with V2 75 cycles (75-bp Single Read). Reads were quantified using *kallisto* (Bray et al., 2016) with the honey bee transcriptome (version Amel_HAv3.1) as a reference, using default parameters. The R package DESeq2 was used to normalize and determine which genes were differentially expressed among control and treatment samples, setting the control group as reference to be compared against. Genes were considered differentially expressed at an FDR adjusted *value of p* <0.05. To visualize the differences in expression profile between the samples, the *plotPCA* function in DESeq2 was used to generate principal component analyses. MA plots visualizing base-2 log fold-change (LFC) (y-axis) versus normalized mean expression (x-axis) in the tetracycline treatment against the control were plotted using the *ggmaplot* function on previously shrinked effect sizes using the *lfcShrink* function for better visualization and ranking of genes. To study the amount by which each of the significantly different determined genes deviates in a specific sample from the gene’s average across all samples we created a heatmap using the *pheatmap* function on regularized logarithm *rlog()* transformed data. Gene ontology (GO) enrichment analysis of the significantly differentially expressed genes were carried out using GOstats, GSEABase and Category R packages (Falcon and Gentleman, 2007). Biological processes associated with these GO terms were summarized and visualized using REVIGO (Supek et al., 2011).^1^ The semantic similarity was measured using the Resnik’s measure (SimRel) (Resnik, 1999) and the threshold used was C = 0.7 (medium). The results were then used to produce a scatter plot using the ggplot2 package in R.

## Results

### Microbiomes affect bee immunity and survival under high toxin stress

**Figure 3:**
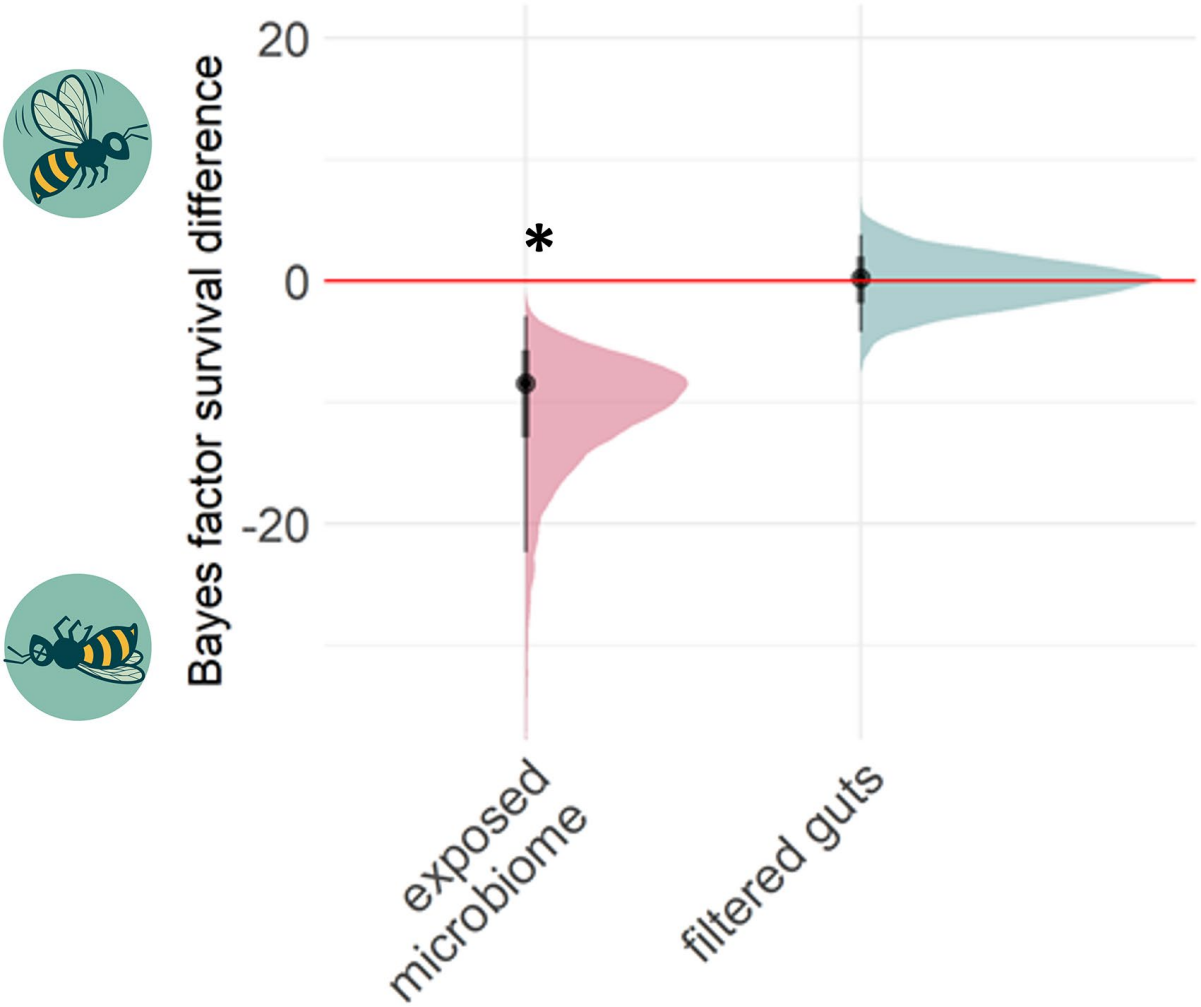
Past chemical exposure of a microbiome can affect future host survival. The Bayes factor difference between treatment and control groups measures whether survival in treatments was higher (positive axis) or lower (negative) under a high dose of tetracycline relative to the respective control group. 95% posterior distribution confidence intervals lying outside zero are highlighted by asterisks. Transferring tetracycline pre-exposed guts (*N* = 3 cages; altogether 53 control-gut and 47 tetracycline-gut individuals) negatively affected bee health under high stress, while transferring filtered gut solution together with a healthy adult microbiome (*N* = 4 cages; altogether 60 control-filter-gut and 50 tetracycline-filter-gut individuals) did not affect the survival.

Bee guts were transferred three times to new hosts after exposure to sub-lethal doses of tetracycline. Bee survival during the 3 cycles showed higher mortality under tetracycline in all cycles in comparison to respective control (Supplementary Figure S2). At the end of these transfers, in cycle 3, naïve recipient bees were given lethal doses of the tetracycline. Survival was compared between bees receiving chemical-exposed microbiomes and those receiving unexposed control microbiomes. The microbiomes with previous tetracycline exposure significantly decreased the survival of the host bees (Bayes Factor (BF) comparing survival in control vs. dysbiotic treatments 95% CI -26.84 – −4.38) (−34% survival, *p* < 0.001, *Fisher’s exact test* on alive/dead count data) (Figure 3).

We further experimentally investigated if the microbiome itself or rather tetracycline residues inside the transferred guts affected the bee survival. We found no support for the latter hypothesis, as the filtered gut solutions did not decrease survival under high stress (BF 95% CI -4.14 – 3.88) (*Fisher exact test*, *p* = 0.64).

### Tetracycline affects the bacterial community composition

Challenging bees with tetracycline over two cycles (“worker generations”), affected microbial community composition. We examined the gut microbial community composition of 54 individual bees from four different time points during the experiment as well as six hive nurse bees, the start microbiome, 12 microbiome transfer samples and two mock DNA controls. The V3-V4 region of the bacterial 16SrRNA gene was amplified and sequenced on the Illumina MiSeq platform, generating an average of 30,462 reads per sample (range, 14,253 to 65,293). The total number of ASVs was reduced from 1717 to 460 after filtering out mitochondria, chloroplasts, artifacts and reads not assigning to the kingdom Bacteria. The two mock community control DNA samples (ZymoResearch cat D6306) sequenced in this study showed no qualitative differences compared to expected theoretical proportions provided by the mock community manufacturer (Supplementary Figure S3). ASVs matching non-mock taxa belonged to honey bee core symbionts but accounted for only 0.23% of the abundance, representing neglectable cross-contamination during library preparation or sequencing. Rarefaction plots on the minimum sample count (Supplementary Figure S4) show quickly reaching converged lines in all samples, indicating sufficient depth. We observed no significant differences between whole bee and microbiome transfer samples for control as well as tetracycline treatments (PERMANOVA; control: *p* = 0.52, *R*^2^ = 0.03, *F* = 0.75; tetracycline: *p* = 0.55, *R*^2^ = 0.03, *F* = 0.72). Based on these results we continued analyzing the transfer and bee samples together.

**Figure 4:**
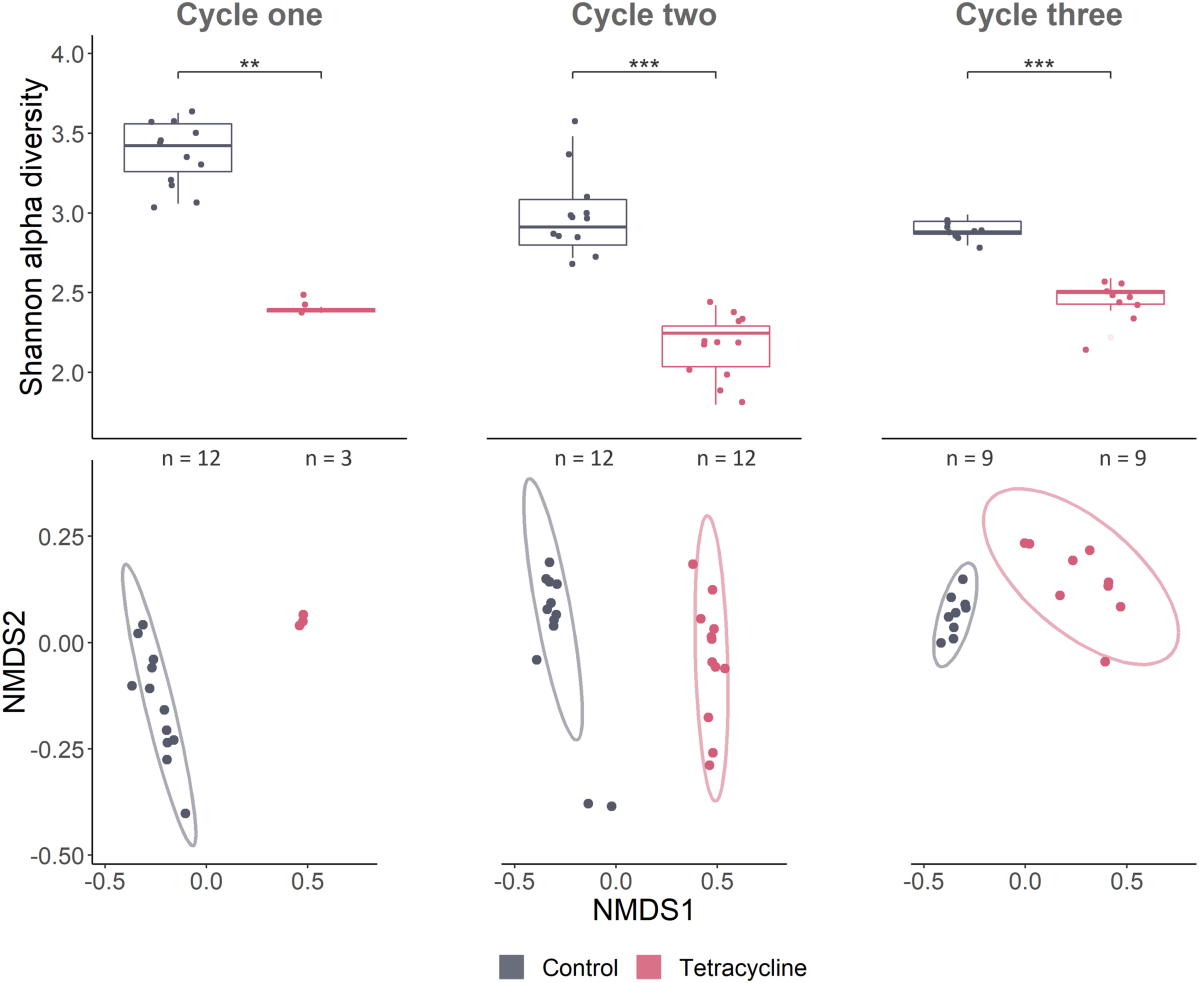
Gut microbial community composition responds to tetracycline treatment. Alpha- and beta-diversity as well as taxonomy show tetracycline leading to a strong dysbiosis, decreasing several taxa. Alpha diversity Shannon index accounts for abundance and evenness of ASV in samples. Pairwise Wilcoxon rank sum tests were used for statistical comparisons between treatments and controls (*** < *p* 0.001; ** < *p* 0.01) (cycle 1: *W* = 36, *p* = 0.004; cycle 2: *W* = 144, *p* < 0.001; cycle 3 before stress: *W* = 81, *p* < 0.001). NMDS on Bray–Curtis dissimilarity which considers presence/absence as well as abundances of ASVs, represents compositional differences between samples (beta diversity). Stress of NMDS was 0.069. Ellipses represent 95% confidence intervals around treatment centroids.

Microbial alpha diversity was much lower in the tetracycline treated individuals at all time points, as measured with the Shannon index (Figure 4) and numbers of observed species (Supplementary Figure S5). This effect could be seen using Non-metric Multidimensional Scaling (NMDS) with tetracycline-treated samples being distinct from control samples (Figure 4). PERMANOVA on Bray-Curtis distances identified tetracycline-stressed microbiomes as being significantly different from controls (cycle 1: *p* = 0.003, *F* = 31.2, *R*^2^ = 0.71; cycle 2: *p* < 0.001, *F* = 62.5, *R*^2^ = 0.74; cycle 3; *p* < 0.001, *F* = 41, *R2* = 0.72). Treatments did show significant effects on groups dispersion in cycle 1 (permutest*; p* < 0.001, *F* = 11.4), and 3 (*p* = 0.01, *F* = 8.5) but not in cycle 2 (*p* = 0.64, *F* = 0.19) (Supplementary Figure S6) indicating that high dispersion may affect the PERMANOVA statistical output.

**Figure 5:**
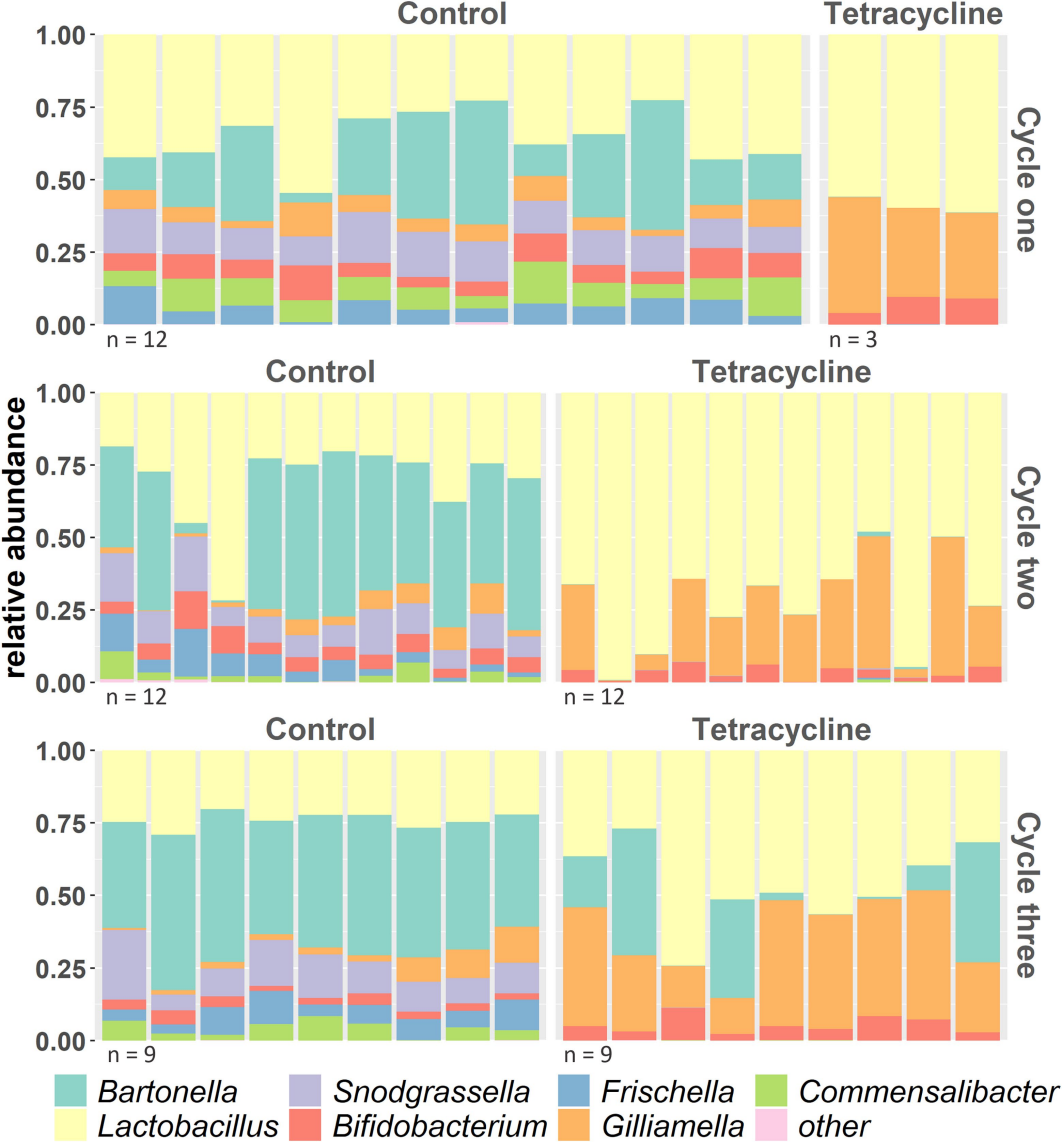
Taxonomy of bacterial genera across the 3 cycles (cycle three before high stress application) with at least 1% relative abundance across samples (everything else is combined to “others”) shows several taxa disappearing under tetracycline.

At the end of the first cycle, several bacterial core genera disappeared from guts of antibiotic-fed bees, namely *Frischella, Bartonella, Snodgrassella* and *Commensalibacter* (Figure 5). The abundances of almost all core symbionts were significantly affected by tetracycline (Supplementary Figure S9 and Supplementary Table S1 for stats). On a finer scale, we observed in several bacterial species some ASVs being susceptible to antibiotic treatment and getting eliminated, while others were unaffected or even increased in relative abundance (Supplementary Figure S10).

### Tetracycline affected microbial communities affect host gene expression

We sequenced mRNA of one honey bee per cage (three per treatment and control respectively) in cycle three before high stress application, with an average of 99.5 million (min 4.8 million, max 567 million) raw reads. While most of these reads mapped to bees, the pathogen Nosema could be detected as a higher percentage of the control reads (0.35, 0.95, 0.11 percent aligned) in comparison to the tetracycline treated bees (0, 0, 0.04 percent aligned) in the taxonomy analysis of NCBI on the submitted raw reads. The pseudoalignment rates of the samples were 64 土5.1% (s.d.).

**Figure 6:**
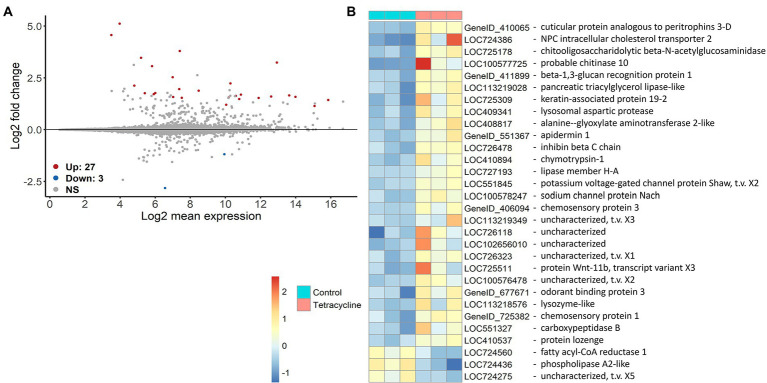
Differential gene expression of genes in naïve bees that received tetracycline-exposed in comparison to individuals that received control microbiomes. MA plots show the differential expression of the tetracycline-gut against the control-gut treatment (*n* = 3 (one bee per cage for both treatments respectively)) **(A)**. The x axis shows the average expression over the mean of normalized counts, and the *y* axis shows the gene-wise dispersion estimate’s shrunken log2 fold change. Red and blue points indicate significant up- or downregulation (FDR ≤ 0.05 determined by DESeq2) of individual genes. Heatmap on *rlog()* transformed data shows the expression difference of each significantly different gene in a specific sample from the gene’s average across all samples. In addition the gene descriptions are shown **(B)**.

Differential gene expression analysis showed that receiving the antibiotic-disturbed microbiomes affects host gene expression. Altogether 30 genes were significantly differently expressed (*p* > 0.05) after FDR adjustment for multiple comparisons (Figure 6). Surprisingly, only three genes were down-regulated and are mainly involved in lipid metabolism such as *phospholipase A2-like* (LOC724436) and *fatty acyl-CoA reductase 1* (LOC724560). Some of the up-regulated genes have likely functions in immunity such as *apidermin 1* (GeneID_551367) or *lysozyme-like* (LOC113218576), transport activities, e.g., NPC intracellular cholesterol transporter 2 (LOC724386) and metabolism such as *lipase member H-A* (LOC727193) or *chitooligosaccharidolytic beta-N-acetylglucosaminidase* (LOC725178) (Figure 6 and Supplementary Figure S11). As we sequenced only three individuals per treatment it is important to be cautious about generalizations. However, while the tetracycline-pre-treated-gut bees showed more within-group variation in their expression profiles comparison to the control (Supplementary Figure S12), the significantly different genes showed relatively similar expression patterns within the two groups although both coming from three individual cage communities (Figure 6). See additional information such as the lists of up- and down regulated genes with information on gene description, GO term and beebase IDs in the Github folder.

## Discussion

Considering the worldwide increase in variety and abundance of anthropogenic stresses together with the loss of biodiversity (Barnosky et al., 2011, 2012), there is urgent need to understand all potentially contributing effects. This includes consideration of interactions between organisms. How microbiomes affect host’s responses to such selection remains underexplored (Cavicchioli et al., 2019). Associated microbial symbionts and their functional relationships with their hosts are sensitive to disturbance. Given that microbiomes are vertically inherited, wholly or in part, in many organisms, any changes in composition and associated second-order effects on organismal health may be propagated across generations.

Here, we used controlled lab experiment to show that deleterious effects of antibiotics on the microbiome can be passed across generations and affect host health decoupled from any direct toxicity of the antibiotic.

### Antibiotics reduce microbiome diversity on genus- species- and strain level

Consistent with the direct action of antibiotics on bacteria, we observed substantial changes in the honey bee gut community after tetracycline exposure. While in previous studies, antibiotics were shown to affect the honey bee microbiome (Powell et al., 2021; Tian et al., 2012; Moullan et al., 2015; Li et al., 2017; Raymann et al., 2017; Baffoni et al., 2021; Jia et al., 2022), it rarely led to the total collapse of bacterial species as we observed in our design. At the end of the first cycle, four bacterial genera disappeared from guts of antibiotic-fed bees (Figure 5 and Supplementary Figure S9). In general, it may be difficult to compare different studies as they differ in methodology. Also, honey bees used in the studies may differ genetically, in their surrounding environment and likely in their colony’s chemical exposure histories which can affect the microbial strain composition (Tian et al., 2012; Ellegaard and Engel, 2019; Wu et al., 2021). Low antibiotic intake (10 ug/mL) after emergence did not show to affect the later establishment of the microbiome in honey bees (Jia et al., 2022). However, we used previously published higher concentrations (Raymann et al., 2017) which were administered immediately after emergence, which could affect the uncolonized gut environment and the overall response of a microbiome. Cox et al. introduced early life as “critical developmental window” when antibiotics have greatest impact on the gut microbiome of mice, leading to lasting metabolic consequences (Cox et al., 2014). Such time-dependent response difference has also been demonstrated in honey bees (Motta and Moran, 2020), which eclose largely without microbes and then acquire them from the surrounding environment and nestmates (Powell et al., 2014). After the high tetracycline exposure in cycle three, the microbiome composition did not change (Supplementary Figure S7). For the tetracycline-treated microbial communities this could be explained by the fact that we selected for antibiotic resistant strains in the previous cycles, however we also did not observe changes in the controls. While this seems surprising considering the extreme effects of lower antibiotic dosages in the cycles before, this may be caused by the fact that we firstly applied tetracycline to fully colonized adults in cycle three and secondly that we sampled 20 h after antibiotic exposure and DNA sequencing will also capture dead material. Other studies also detected a more prominent effect of antibiotics on the honey bee gut community several days after treatment was stopped which may be a result of a delayed effect of the antibiotic (Raymann et al., 2017). In addition, while 16S sequencing has limitations when it comes to fine-scale taxonomic identification (Ellegaard and Engel, 2016), we found extensive response variation at the generic, species, and ASV levels (Supplementary Figure S10). This is consistent with other studies that found effects of antibiotics (Raymann et al., 2018) and other pesticides (Cuesta-Maté et al., 2021) vary across bee gut bacterial species and strains. These data together with the increase in resistance genes in antibiotic exposed bee microbiomes (Tian et al., 2012; Sun et al., 2022) indicate adaptation to chemical selection factors.

### Negative effects of antibiotic-disturbed microbiomes can be transferred to following generations

In general, perturbations of a healthy gut environment can affect gene expression, protein activity, and the overall metabolism of a host associated gut microbiota (Franzosa et al., 2015). Antibiotic exposure causes dysbiosis, with effects on host health (Francino, 2016; Neuman et al., 2018), the resistome (genes involved in resistance responses), and gut bacterial diversity (Li et al., 2019; Xu et al., 2020). We found that short-term dysbiosis could be transferred to subsequent worker bee generations which is in line with previous experiments in honey bees and flies (Ourry et al., 2020; Jia et al., 2022). In our experiment, tetracycline disrupted the normally stable bee gut community, which did not recover over subsequent generations even after antibiotic administration was ceased. In cycle three only *Bartonella* could recover in some samples, while the other antibiotic-affected genera appeared permanently eliminated from the community (Figure 5). This transmitted dysbiosis was likely the reason of the higher mortality under subsequent tetracycline stress at the end of the experiment in naïve bees that inherited the disturbed microbiome (Figure 3).

Feeding macerated honey bee guts to other bees is an established method of microbial transfer in laboratory studies (Powell et al., 2014; Zheng et al., 2018; Kowallik and Mikheyev, 2021). However, since bees do not defecate in captivity, toxins such as tetracycline could conceivably accumulate in the hindgut. It is therefore imaginable that small amounts of left tetracycline or derivates may negatively affect the health of the following worker bee generation. We excluded this possibility by an additional experiment transferring tetracycline-exposed guts filtered to remove bacteria and seeing no effects on mortality (Figures 2, 3). This supports the interpretation that the detrimental effect was indeed caused by the disturbed microbial community. In general, we cannot exclude that we also transmitted non-bacterial pathogens during microbiome transfer in our design which may affect host health. For the fungal pathogen Nosema a potential correlation has been reported between infection load and gut microbiome structure (Rubanov et al., 2019). However, we do not see a higher Nosema load in the antibiotic treated bees in our RNA data but rather the opposite. In addition, none of the significant genes in our design are common pathogen-response genes. The humoral immunity in honeys bees involves synthesis of antimicrobial peptides (AMPs) from which *abaecin*, *apidaecin*, defensin and *hymenoptaecin* usually respond to bacterial, viral and fungal infection (Evans et al., 2006; Chaimanee et al., 2012; Flenniken and Andino, 2013; Doublet et al., 2017).

Gut bacteria function as a protective barrier, enhancing nutritional provisioning and affecting the host immune system across animal systems (Hooper et al., 2012; Tremaroli and Bäckhed, 2012; Kamada et al., 2013) including honey bees (Kešnerová et al., 2017; Raymann and Moran, 2018). Administration of antibiotics has been shown to reduce gene expression of antimicrobial peptides in bees (Li et al., 2017; Motta et al., 2022). We observed a significant up-regulation of genes having functions in immunity, biotic responses, carbohydrate metabolism and transport for all kind of molecules (e.g., metal ion, sodium ion, sterol transport) in bees receiving dysbiotic microbiomes (see Figure 6 and Supplementary Figure S11). Only three genes showed to be down-regulated which were mainly involved in lipid metabolism. As our cycle three bees did not consume tetracycline themselves, we can conclude that the differential gene expression was most likely caused by the microbial community changes. Changes in community structure such as those observed in our study can alter the provided microbiome function such as provision of nutrients or removal of toxic metabolites across systems (Willing et al., 2011). In general, interactions between symbionts can be as important as the individual species in gut microbiomes, therefore the effects of a disturbed microbiome go far beyond the loss of functions attributable to single taxa (Gould et al., 2018). In our design, a disturbed cross-talk between host and microbiome could have affected host gene expression as the host may have had to compensate for missing functions. However, as we sequenced only one bee per cage and the expression of bees receiving tetracycline pre-exposed microbiomes shows higher within treatment variation than the control (Supplementary Figure S12) we should be cautious with generalizations.

### The honey bee as model system

Previous work characterized the honey bee microbiome and developed methods such as artificial microbiome transmission (Engel et al., 2013; Powell et al., 2014; Kwong and Moran, 2015). We built on this foundation using honey bees as a model to study stress-induced, microbiome-mediated effects on subsequent generations. In our experiments we performed a purely vertical microbiome transfer between individuals, a rate at the extreme end of a continuum of strategies. While in most systems microbes are acquired both vertically and horizontally, high rates of vertical transfer are typical in honey bees (Engel and Moran, 2013). We did not provide the opportunity to recruit different strains through the environment or social contact inside the hive which could have led, for instance, to some recovery from the dysbiotic state induced by tetracycline or could have led to colonization of opportunistic pathogens. Although a previous study did not find that honey bees with antibiotic-induced dysbiosis recovered their microbiomes to a healthy state when being put back to the hive environment and that they also suffered from higher mortality in this natural environment compared to the control (Raymann et al., 2017). Beside chemically induced changes to the microbiota, even communities in our control treatment were also gradually changing in the lab. For instance, we observed an increase of *Bartonella* abundance in all treatments in comparison to hive nurse siblings and the starting microbiome pool (Supplementary Figure S8). These changes likely reflect lab adaptations and emphasize the need to run proper lab controls in microbiome experiments (Arora et al., 2020), but also a need to run more natural experiments in the future. Additionally, the high tetracycline dosage over two worker generations may not reflect natural conditions, though mimicking nature was not our intent.

Controlled laboratory experiments such as microbiome transplants, provide the most convincing insights into functional host-microbiome relationships (Greyson-Gaito et al., 2020). They are invaluable because they can simplify the complexity and disentangle factors to achieve fundamental understanding which is still lacking in the field. However, these experiments trade control for natural complex conditions, which is important for drawing ecological and evolutionary conclusions (Carrier and Reitzel, 2017).

In addition to being a tractable model for microbiome research, honey bees are important pollinators in natural and agricultural ecosystems (Hung et al., 2018). They are exposed to diverse agricultural chemicals including those applied to plants making up their diet but also the ones used by beekeepers to prevent infection or suppress parasites (Ortiz-Alvarado et al., 2020). Antibiotics have been experimentally demonstrated to disturb the core microbial bee microbiome, lowering diversity on species and strain level and leading to negative health effects (Raymann et al., 2017, 2018; Powell et al., 2021; Jia et al., 2022). Facilitated by social transmission between workers, changes in the microbiome could theoretically quickly go to fixation in a population. Indeed, antibiotic resistance genes have accumulated in bacterial symbionts in managed honey bee colonies, demonstrating long-term impacts with unknown consequences (Tian et al., 2012; Ludvigsen et al., 2017; Daisley et al., 2020; Piva et al., 2020). Considering the social-vertical transfer of the microbiome between worker generations in honey bee colonies with the fact that chemicals including antibiotics accumulate and persist in the hive environment over longer periods (Martel et al., 2006), the damage on the bee microbiome could theoretically go beyond one individual’s health affecting a whole population. In mice, diet-induced progressive loss of taxonomic diversity is cumulative over generations and indicate that taxa driven to low abundance are inefficiently transferred to the next generation, and are at increased risk of becoming extinct within an isolated population making this change eventually irreversible (Sonnenburg et al., 2016). This suggests that multigenerational environmental exposure could indeed cause a stable transgenerational alteration of organism physiology *via* the microbiome.

## Conclusion

Co-evolved microbiomes can offer a range of benefits to their hosts and vice versa. However, under disturbance this picture may change, and the dependent partner could suffer negative consequences. While it is often difficult to disentangle cause and consequences of chemical-induced microbiome disruption on host health, we provide evidence that a disturbed microbiome and its mediated effects on host phenotypes can get transmitted across generations in a lab environment. This “dark side” of a specialized, vertically transferred microbiome could, likewise as negative mutations, theoretically go into fixation affecting the health of a whole population if no refreshing is possible. This is particularly true if the whole population is affected by chemical stress, for example in an agricultural context. For instance, agrichemical degradation of microbiomes may be a plausible, silent factor underlying global insect declines. Future studies would be important to examine the extent to which negative microbiome-mediated phenotypes are really heritable in the field. Examining whether such heritable dysbiosis has the potential to threaten host populations or which potential rescue mechanisms may play a role to prevent such scenario under natural conditions would be relevant to further understand organism health and conservation.

## Data availability statement

The datasets presented in this study can be found in online repositories. The names of the repository/repositories and accession number(s) can be found at: https://www.ncbi.nlm.nih.gov/, PRJNA863631. All data files and codes used for processing and analysis can be found following this link: https://github.com/kowallik/inheritance_microbiome_disturbance. RNA and 16S raw reads are available under NCBI Bioproject PRJNA863631.

## Author contributions

VK and AM wrote the manuscript. AM processed raw RNA sequences. VK designed research, conducted experiments, processed 16S sequences, and analyzed 16S, RNA and survival data. AD extracted samples and prepared RNA libraries. All authors contributed to the article and approved the submitted version.

## Funding

A fellowship provided by the German Research Foundation (DFG) (KO 5604/1–1) and the Okinawa Institute of Science and Technology (OIST) supported VK’s research. AM was funded by a Future Fellowship from the Australian Research Council (FT160100178).

## Conflict of interest

The authors declare that the research was conducted in the absence of any commercial or financial relationships that could be construed as a potential conflict of interest.

## Publisher’s note

All claims expressed in this article are solely those of the authors and do not necessarily represent those of their affiliated organizations, or those of the publisher, the editors and the reviewers. Any product that may be evaluated in this article, or claim that may be made by its manufacturer, is not guaranteed or endorsed by the publisher.

## Footnotes

^1^ http://revigo.irb.hr
