# Supplementary material for: Experimental inheritance of antibiotic acquired dysbiosis affects host phenotypes across generations
Source: Front Microbiol. 2022 Dec 1;13:1030771. doi: 10.3389/fmicb.2022.1030771 (PMC9751584; doi:10.3389/fmicb.2022.1030771)
Supplement: Supplementary file 1 [file Data_Sheet_1.PDF]

Supplementary Material

1 Supplementary Data

Main experiment additional details

At the start of the experiment, frames from one honey bee colony located at ANU, Canberra, Australia were moved into the lab. All material (tools, cages, cotton etc.) has been autoclaved and/or UV sterilized prior usage. Late-stage pupae with black eyes but lacking movement were carefully taken out of the frames using soft forceps and placed on cotton in plastic containers (Figure S1). During the whole procedure, forceps were flame-sterilized in between using ethanol and Bunsen burner and a face mask was worn to prevent contamination. The containers were placed in a clean desiccator inside an incubator at 35 degrees and ~75% humidity (boosted with K_2SO_4 salt solution (1). Bees emerging within a time frame of 24 hours were randomly distributed into six sterile self-crafted plastic cages (Figure S1) and fed with filter-sterilized 0.5M sucrose solution for the first hours. Emerged bees were kept at 35 degrees in incubators with ~60% humidity. After bees were distributed, we transferred natural microbiome from hive siblings to them. For that, 10 nurse bees from the same hive were surface sterilized by soaking them in 85% ethanol for 1 minute with three following washing steps in ultrapure water. Guts were dissected, and the hindguts were macerated in 200 µl of an equal mix of PBS buffer and sucrose solution (a bit was saved for later sequencing) with added gamma-irradiated pollen. This mixture was equally distributed between cages. This procedure was repeated after 24 hours with another batch of 10 nurse bee gut transfers. After these first 48 hours of inoculation time, only sterile food was given to the bees for the rest of the experiment. We replaced two feeders per cage every day with freshly prepared pollen and sucrose solution with or without previously published concentrations of 450 µg / mL tetracycline (2) and removed dead individuals. For the microbiome transfer between cycles, three bees per cage were surface sterilized and their hindguts were mixed with pollen and provided as food source to one cage of the next generation for 48 hours. Afterwards the normal feeding as described above was continued.

High toxin concentrations for final functional test in cycle 3

To determine the appropriate concentration of tetracycline for the final functional test, we used nurse bees from the same colony and tested three different concentrations to compute ~LD50 (concentrations under which ~50% of exposed honey bees die after 24 hours). We used one cage with ~25 bees per concentration and ran two control cages without chemicals as negative controls. We used rounded 50, 100 and 500 times (20 mg, 40 mg and 200 mg tetracycline per mL respectively) the concentrations used during the first two experiment cycles and checked the cages after 24 hours. We saw no mortality in the control cages. Under tetracycline ~50% of bees died for the 50 as well as 100 times concentration treatments (while the ones alive in the latter were more dead than alive) and every bee was dead with the highest concentration. Therefore, we decided to use a tetracycline concentration of 20 mg / mL.

Species and ASV details

To further explore taxonomic diversity and response variances in our samples, we examined the species and ASV levels of the core genera (Figure S10). To not only rely on the SILVA database output, we extracted the most abundant ASVs (>1000 reads) for each genus and blasted them online against the full NCBI Nucleotide collection database (high similarity, megablast search) which could verify the

available and add species information. The species in the core microbiota set are: *Frischella perrara* (three ASV), *Bifidobacterium asteroides* (three ASV), *B. coryneforme*/*B. indicum* (one ASV, 100% identity to both species), *Gilliamella apicola* (seven ASV), *Bartonella apis* (three ASV), uncultured *Bartonella* (one ASV), *Commensalibacter sp.* / uncultured *Gluconacetobacter sp.* (three ASV) and *Snodgrassella alvi* (six ASV). Lactobacillus showed to be the most diverse genus with the species hits: *L. melliventis* (nine ASV), *L. mellis* (eight ASV), *L. kullabergensis* (four ASV), *L. apis* (three ASV), *L. kimbladii* (three ASV), *L. kunkeei* (one ASV) and *L. helsingborgensis* (one ASV). Plotting the species abundance across the treatments for *Lactobacillus* shows a difference in response under the antibiotic. The factor cycle showed to not influence the abundance pictures and therefore, we pooled the data from the cycles 1-3 for plotting.

For each *Lactobacillus* species and each of the less diverse other six core bacterial genera, we observed the ASV abundances across the treatments. Under tetracycline there is an obvious between-species variation observed for *Lactobacillus* which is interestingly also shown within species - on ASV level. E.g. from the two abundant ASV for *L. kullabergensis*, one is eliminated under tetracycline while the other one is not affected. For the species *L. apis* only two ASV are low under tetracycline. From the abundant ASVs belonging to the SILVA NA-cluster, the NCBI blast revealed that one ASV belonged to *L. kimbladii*, one to *L. mellifer* eight to *L. melliventris* and another seven to *L. mellis*. Here, five ASV from *L. melliventris* are not detectable anymore under tetracycline, while the other three strongly increase in abundance in comparison to the control. This variation under tetracycline is the same for the *L. mellis* ASVs, with three ASV decrease, two being unaffected and two increase. These response-variations across ASV of one bacterial species could also be observed for *Gilliamella apicola.* From the seven abundant ASVs four were basically not present under tetracycline while being more abundant in the control. For the other three ASVs the opposite was true, they were extremely low abundant in the control but highly abundant in the tetracycline. From the four abundant ASVs belonging to *Bifidobacterium* two ASVs are unaffected and two highly susceptible under the antibiotic.

1. Schmehl DR, Tomé HVV, Mortensen AN, Martins GF, Ellis JD. Protocol for the in vitro rearing of honey bee (Apis mellifera L.) workers. Journal of Apicultural Research. 2016 März;55(2):113–29.

2. Raymann K, Shaffer Z, Moran NA. Antibiotic exposure perturbs the gut microbiota and elevates mortality in honeybees. PLOS Biology. 2017 Mar 14;15(3):e2001861.

2 Supplementary Figures and Tables

2.1 Supplementary Figures

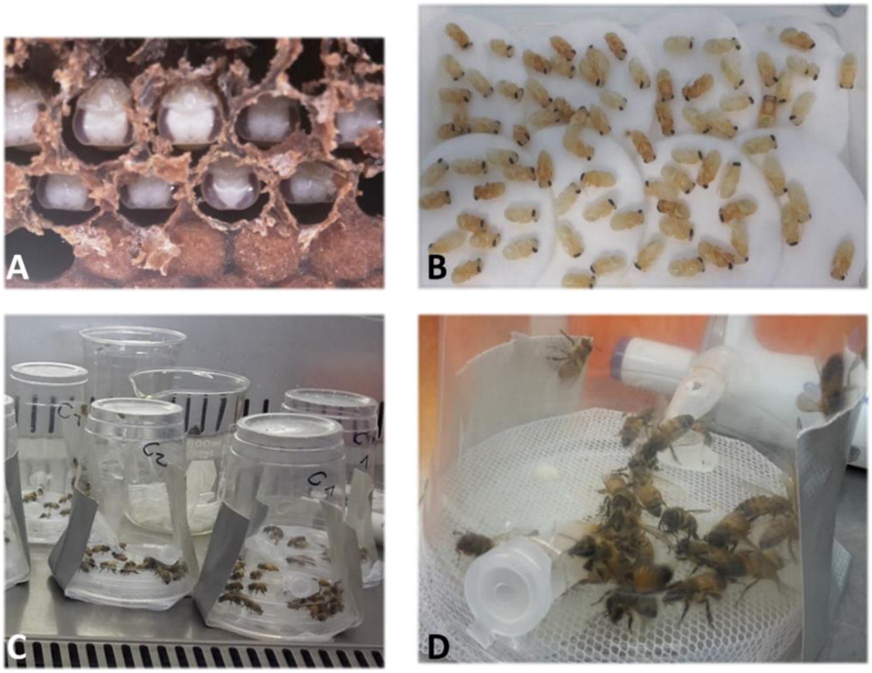

Supplementary Figure 1. Experiment figures. Pupae collected from hive (A) were placed on sterile cotton in sterile container (B). Emerged bees were put in sterile, self-crafted cages (C) where they were walking on mesh (fly net) and had access to two feeders (one for sucrose and one for bee bread) which were replaced daily (D).

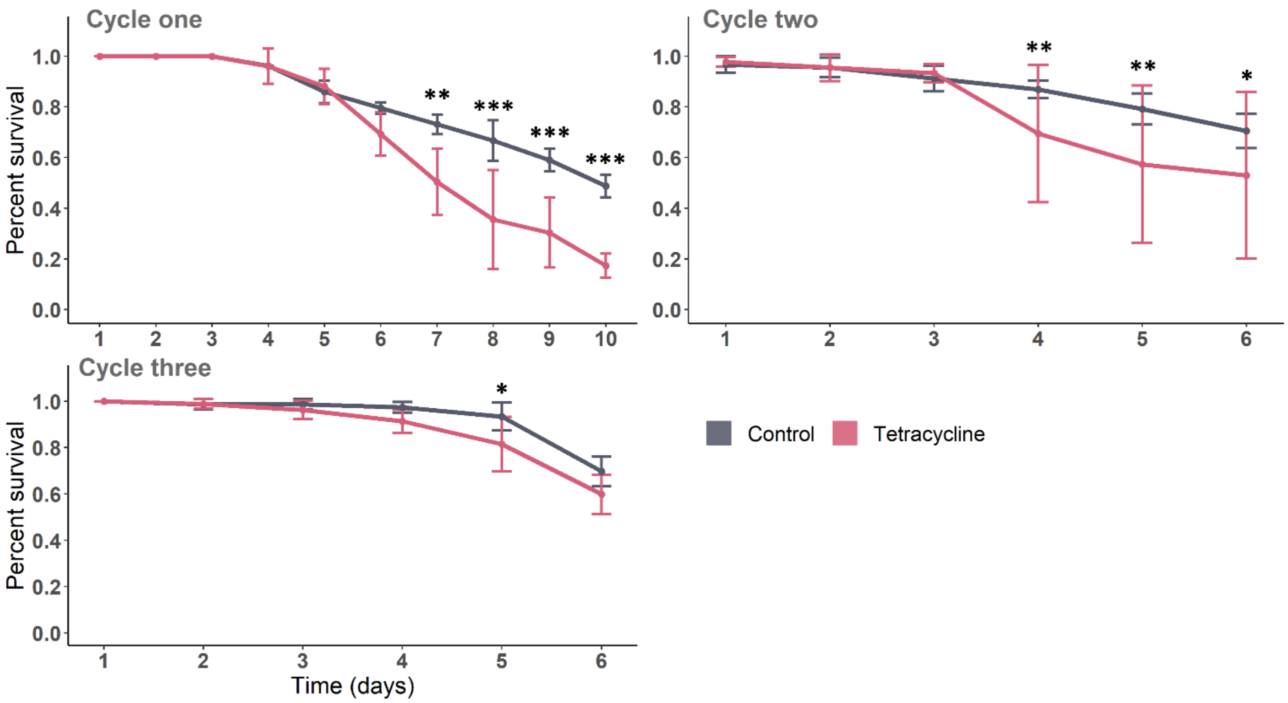

Supplementary Figure 2: Percent survival of bees across the three experimental cycles. Plotted is the mean proportional survival out of three cages (N=3) with added standard deviations. In cycle three only the days before high toxin administration are shown. To compare both treatments two-sided Fisher's exact tests on alive/dead count data of the three cages for each day were performed (*** < *p* 0.001; ** < *p* 0.01; * < *p* 0.5).

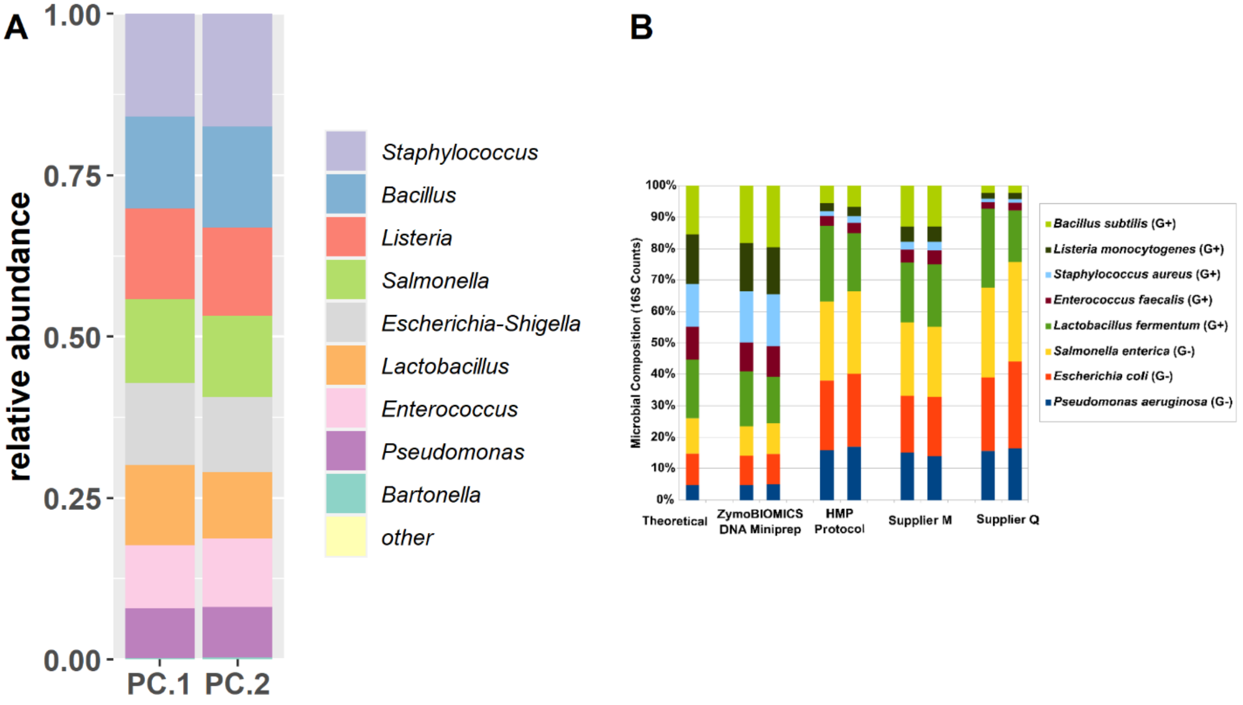

Supplementary Figure 3. The two mock DNA samples sequenced in this study (A) show no big difference to the expected theoretical proportions of the reference (B, bar with label "theoretical"). We plotted taxa accounting for at least 1% of the relative abundance across the samples. In the "other" cluster, we find three non-Mock bacterial taxa belonging to the honey bee core microbiome (*Bartonella*, *Gilliamella* and *Snodgrassella*). Altogether, the relative abundance of these three taxa in the two Mock samples account for 0.23% of all reads, representing neglectable cross-contamination during sequencing.

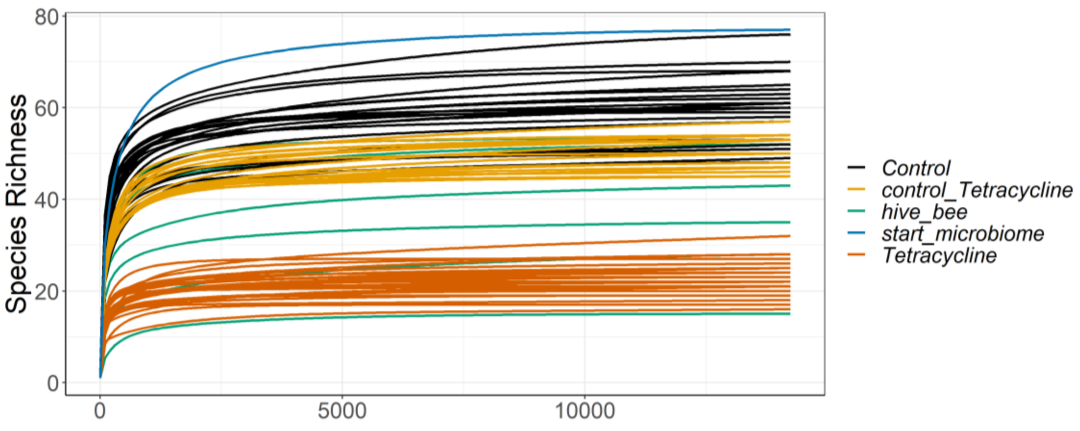

Supplementary Figure 4. Rarefaction curve on the to the minimum depth (14240) standardized, full sample set (Mocks excluded). Rarefaction curves were used as a qualitative method to estimate the species richness as a function of sequencing depth for all samples. They quickly reached their asymptotes for all samples, suggesting that saturation in sequencing was achieved.

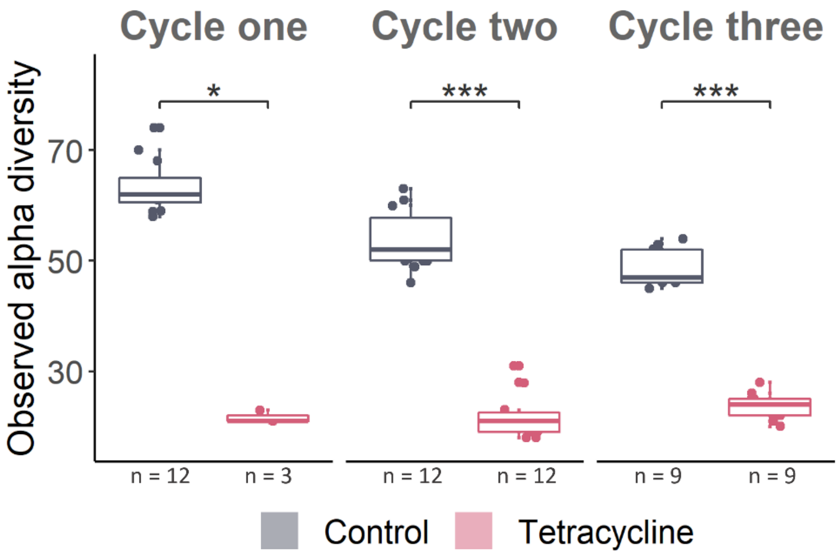

Supplementary Figure 5. Alpha diversity on observed ASVs: The observed alpha diversity plot shows the difference in detected ASVs indicating community richness. Sequences were rarified to minimum depth before and pairwise Wilcoxon-Rank-Sum tests were used to statistically compare the treatments against the respective control (*** < p 0.001; ** < p 0.01; * < p 0.5). In all three cycles tetracycline significantly decreased observed species number (cycle 1: $W = 36$, $p = 0.01$; cycle 2: $W = 144$, $p < 0.001$; cycle 3 before stress: $W = 81$, $p < 0.001$).

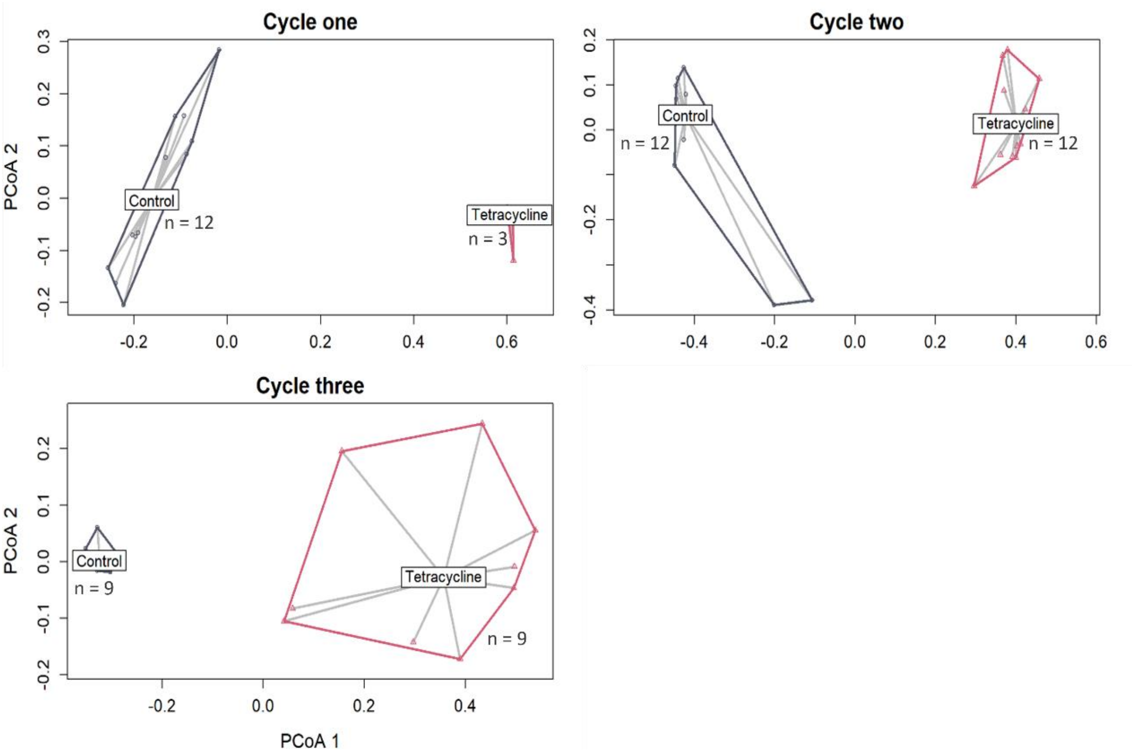

Supplementary Figure 6: Multivariate homogeneity of group dispersions (variances) on proportion data of the samples in cycle 1, cycle 2 and cycle 3 before high stress application. Multivariate tests for homogeneity of variance (with 999 permutations) revealed significant differences between treatments in cycle 1 (permutest*;* $p < 0.001$, $F = 11.4$), while samples were homogenous distributed in cycle 2 ($p = 0.64$, $F = 0.19$) but again significantly different in cycle 3 (p=0.01, F=8.5).

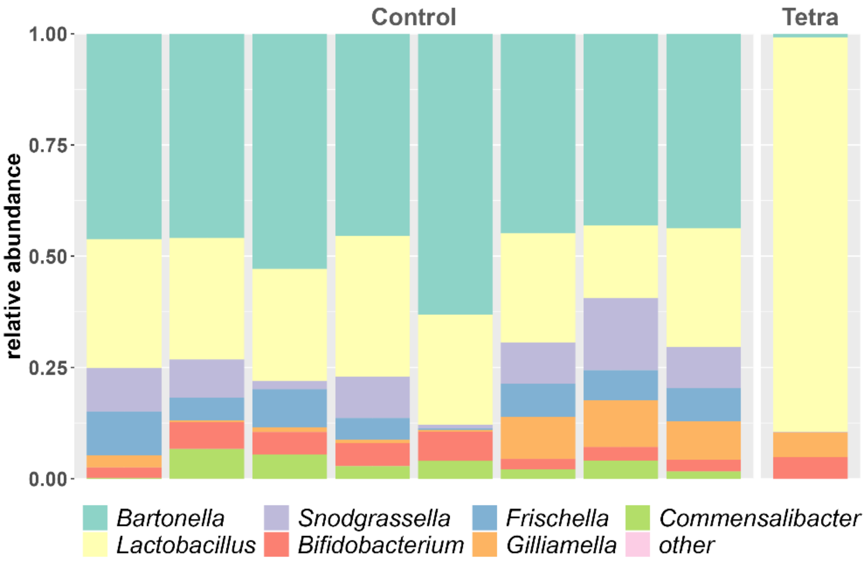

Supplementary Figure 7. Taxonomic output of the samples in cycle three after high tetracycline. Only one bee with the tetracycline pre-treated microbiomes survived. Relative abundance of bacterial taxa on genus level with an abundance of at least 1%. All bacteria with a lower relative abundance are combined into “other”.

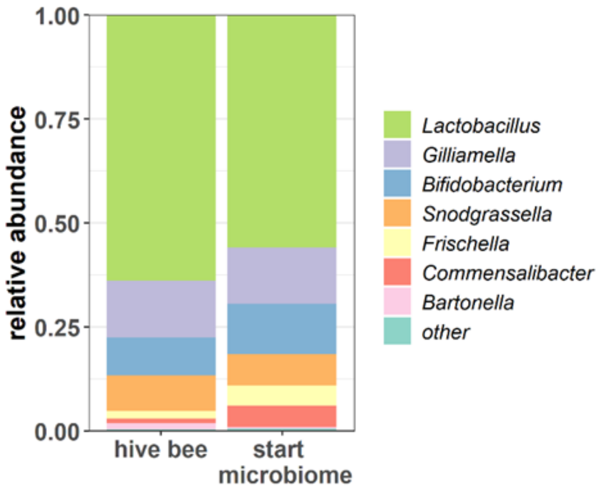

Supplementary Figure 8. Taxonomic output of the hive bee samples (six) and the start microbiome pool used to inoculate all treatments in the lab demonstrates that lab bees during the experiment experienced changes in their microbiome in response to the laboratory conditions (Figure 3) (*e.g. Bartonella* increase). Relative abundance of bacterial taxa on genus level with an abundance of at least 1%. All bacteria with a lower relative abundance are combined into “other”.

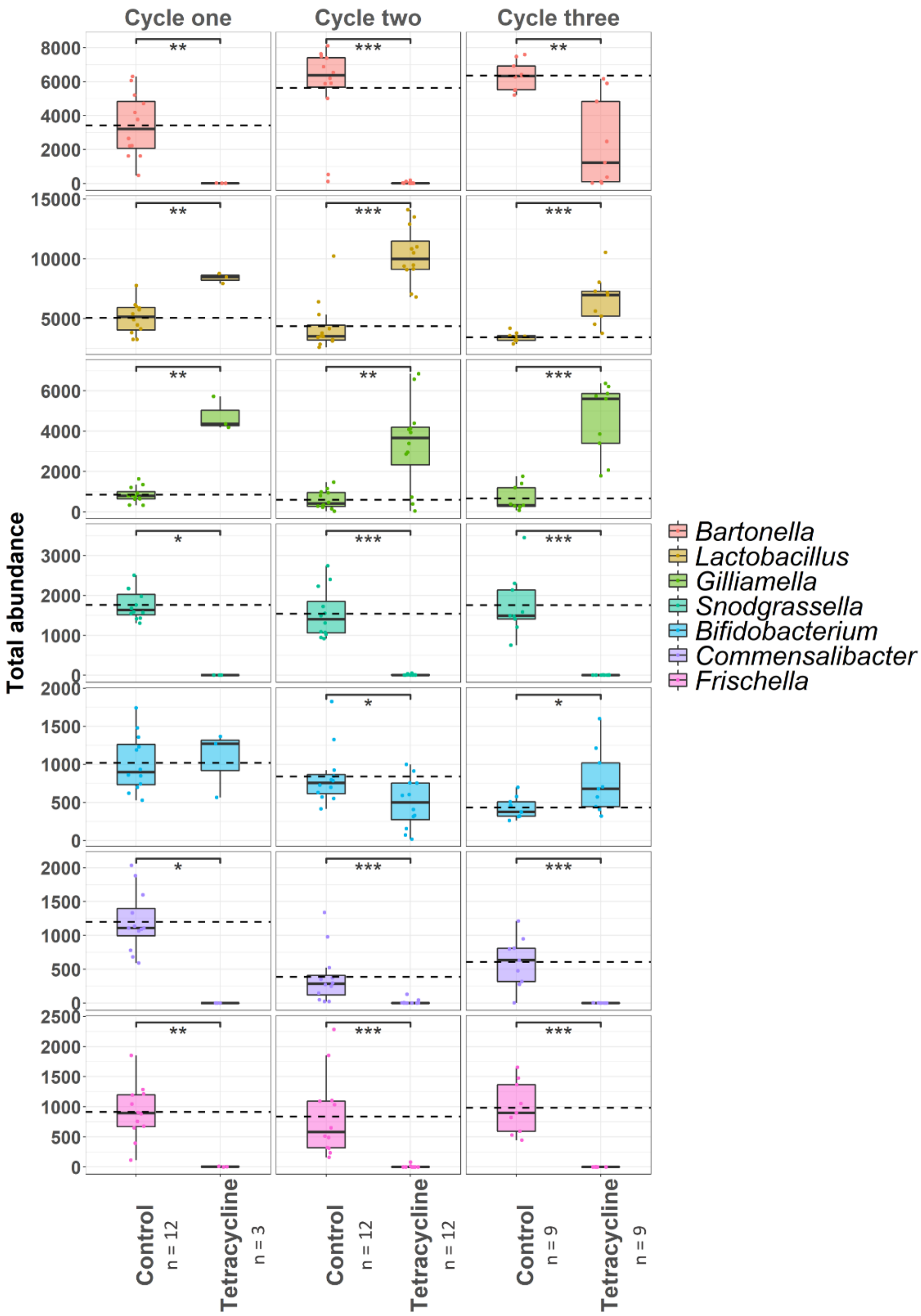

Supplementary Figure 9. Abundance of the seven most dominant bacterial genera across the treatments and the three cycles (cycle 3 = before high toxin application) show that tetracycline changed the abundance of most taxa. While *Bartonella*, *Snodgrassella*, *Commensalibacter* and *Frischella* were decreased to the point of elimination (only *Bartonella* recovers in abundance in cycle 3 when no toxin was applied), *Lactobacillus* and *Gilliamella* increased. Reads have been rarefied to even depth before plotting. Colors indicate the bacterial taxa and the dashed line the mean bacterial abundance of the respective control treatment in each cycle. Pairwise Wilcoxon-Rank-Sum tests with following FDR correction were used to statistically compare the treatments against the respective control (*** < *p* 0.001; ** < *p* 0.01; * < *p* 0.5), see Table S1 for all statistical output.

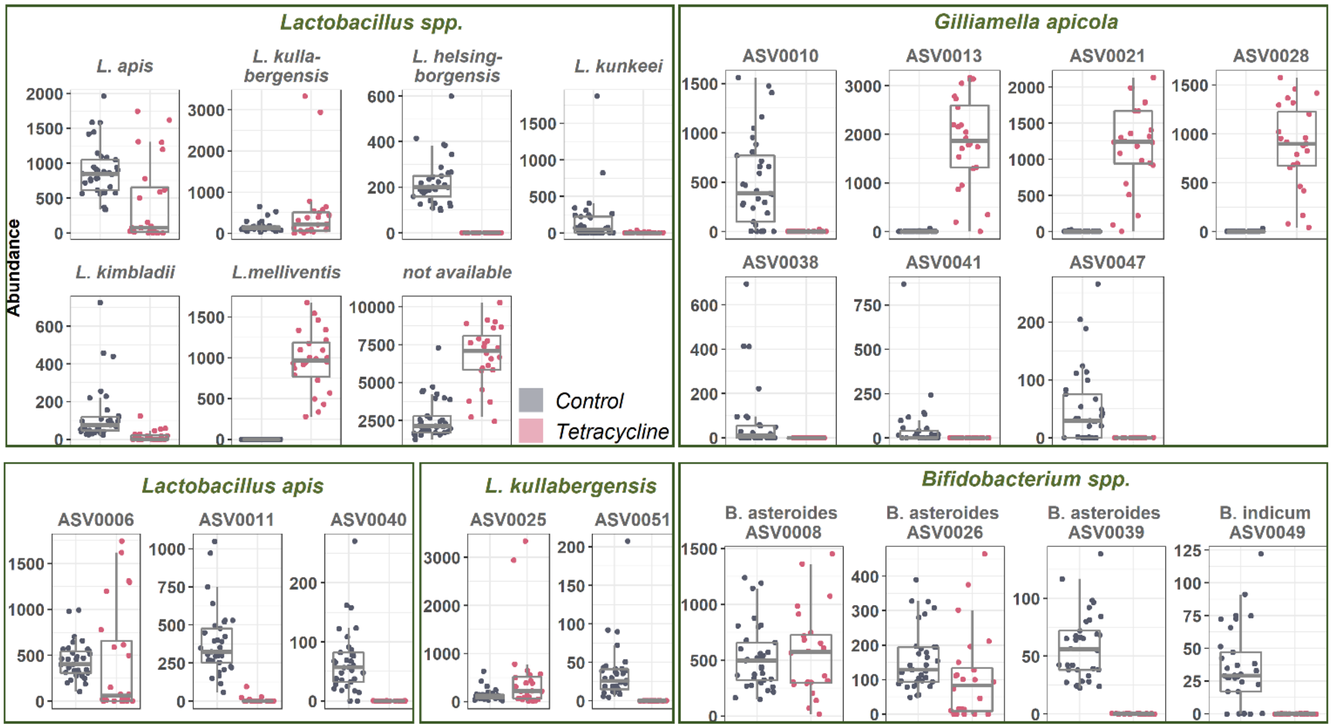

Supplementary Figure 10. Abundance of bacterial species and strains in control (n=33) and tetracycline treated (n=24) microbial communities. Abundance of different *Lactobacillus* species across the treatments show that *Lactobacillus* species respond differently to tetracycline ranging from being eliminated to increased**.** "Not available" indicates that no species level output was available via SILVA database. Blasting these "NA" ASV against the full NCBI Nucleotide collection revealed that these ASVs belonged to *L. melliventris*, *L. mellis*, *L. mellifer* and *L. kimbladii*. Further, response variation existed across strains *in L. apis*, *L. kullabergensis*, *G. apicola* and *B. asteroids*. In general, samples from all three cycles (cycle 1, cycle 2 and cycle 3 before toxin application) are pooled after observing no cycle-specific patterns. Reads have been rarefied to even depth before plotting and only species or ASVs with > 1000 reads are shown. All species names listed are from the SILVA output and re-confirmed by blasting against the full NCBI Nucleotide collection.

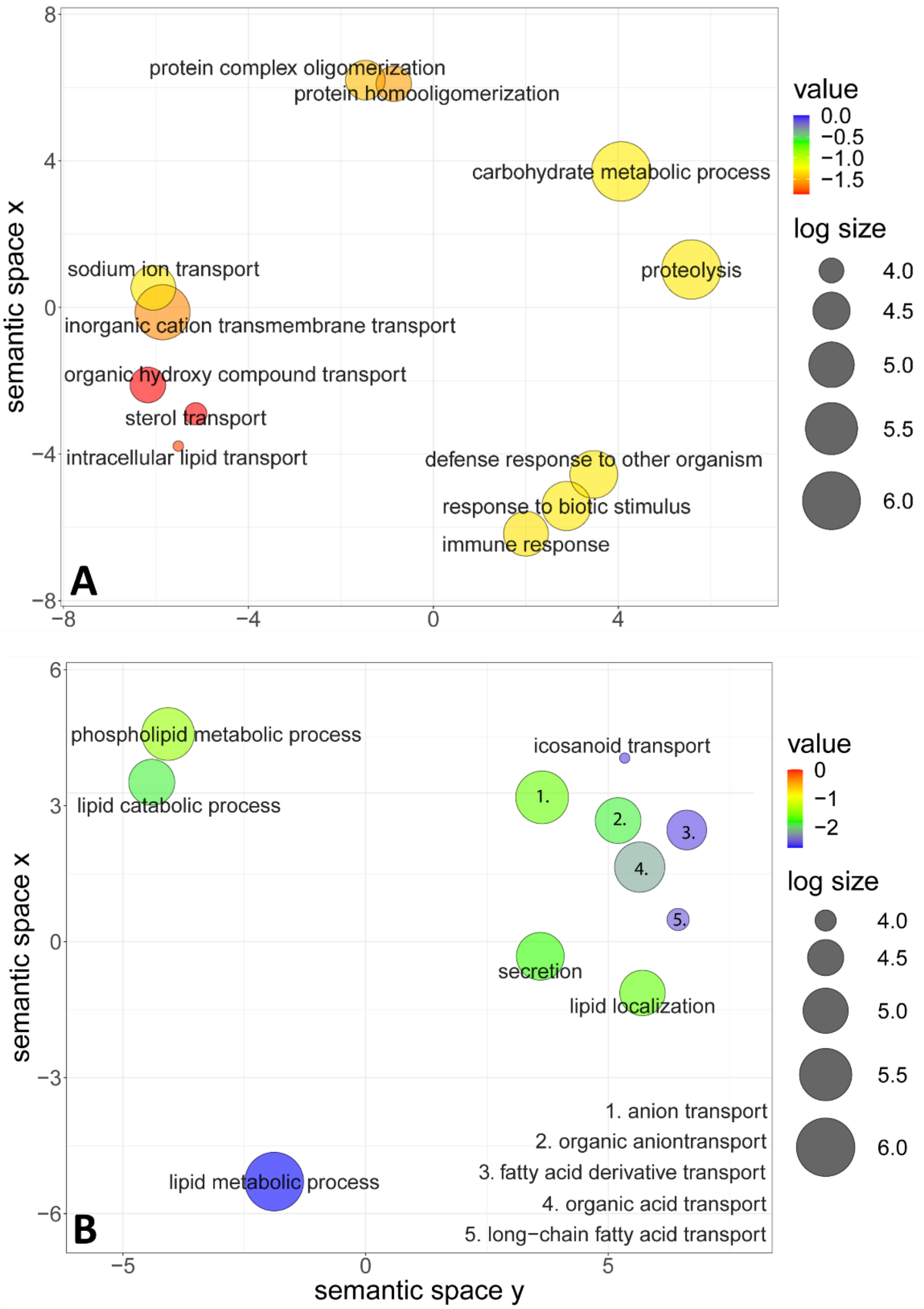

Supplementary Figure 11. GO enrichment analysis summarized and visualized as a scatter plot using REVIGO. (A) Summarized GO terms of the 27 significant up-regulated genes in the tetracycline-treated microbiome bees (N=3) compared to the control-microbiome bees (N=3). (B) Summarized GO terms of the 3 significant down-regulated genes. GO terms are represented by circles and are plotted according to semantic similarities to other GO terms (adjoining circles are most closely related). Circle size is proportional to the frequency of the GO term, whereas color indicates the log10 P value.

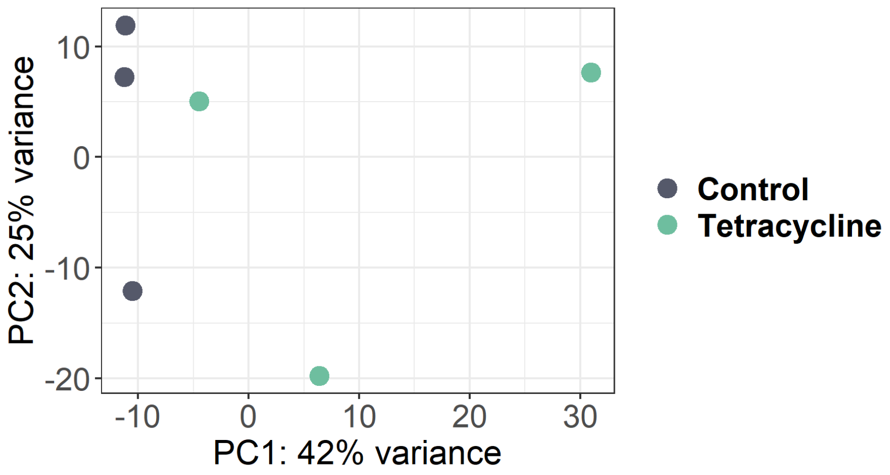

Supplementary Figure 12. RNA expression across treatments. Principal-component analysis (PCA) on RNA data from naïve bees that received tetracycline-exposed or control microbiomes.

2.2 Supplementary Tables

Supplementary Table 1: Pairwise Wilcoxon Tests of each main genus abundance between tetracycline and control treatments for each cycle.

cycle	taxa	W	Wilcoxon *p* value
1	*Lactobacillus*	0	0.004
2	*Lactobacillus*	6	<0.001
3	*Lactobacillus*	2	<0.001
1	*Bartonella*	36	0.004
2	*Bartonella*	142	<0.001
3	*Bartonella*	75	0.001
1	*Gilliamella*	0	0.004
2	*Gilliamella*	22	0.003
3	*Gilliamella*	0	<0.001
1	*Snodgrassella*	36	0.01
2	*Snodgrassella*	144	<0.001
3	*Snodgrassella*	81	<0.001
1	*Frischella*	36	0.004
2	*Frischella*	144	<0.001
3	*Frischella*	81	<0.001
1	*Commensalibacter*	36	0.01
2	*Commensalibacter*	139	<0.001
3	*Commensalibacter*	80	<0.001
1	*Bifidobacterium*	16	0.84
2	*Bifidobacterium*	107	0.05
3	*Bifidobacterium*	17.5	0.05
